# A cross-sectional survey of public knowledge of the monkeypox disease in Nigeria

**DOI:** 10.1186/s12889-023-15398-0

**Published:** 2023-03-29

**Authors:** Ahmad Ibrahim Al-Mustapha, Oluwaseun Adeolu Ogundijo, Nafisah Ayinde Sikiru, Barakat Kolawole, Muftau Oyewo, Hager El-Nadi, Ashiru Mohammed Mustapha, Lucky Icomiare Adebudo, Adesoji Odukoya, Emmanuella Chinenye Asiegbu, Magdalene B. Nanven, Taibat Lawal-Atolagbe, Fatima Lawal-Lah, Oladayo David Awoyale, Ahmed Tijani Abubakar, Kabiru Sahabi, Zaharadeen S. Babandi

**Affiliations:** 1 Department of Food Hygiene and Environmental Health, Faculty of Veterinary Medicine, University of Helsinki, Helsinki, Finland; 2 Department of Veterinary Public Health and Preventive Medicine, Faculty of Veterinary Medicine, University of Ibadan, Oyo State, Nigeria; 3 Department of Veterinary Services, Kwara State Ministry of Agriculture and Rural Development, Ilorin, Kwara State Nigeria; 4 Health Services Department, Federal University, Kashere, Kashere, Gombe State Nigeria; 5 Department of Public Health, Faculty of Basic Medical Sciences, Kwara State University, Malete, Kwara State Nigeria; 6 Nigerian Field Epidemiology and Laboratory Training Program, Abuja, Nigeria; 7 Infectious Diseases and One Health, Faculty of Pharmaceutical Sciences, Universite de Tours, Tours, France; 8 USAID Integrated Health Program, Birnin Kebbi, Kebbi State Nigeria; 9 Edo State Ministry of Agriculture and Food Security, Benin City, Edo State Nigeria; 10 Federal Department of Veterinary and Pest Control Services, Federal Ministry of Agriculture and Rural Development, Abuja, Nigeria; 11 National Hospital, Abuja, Nigeria; 12 Kwara State Ministry of Health, Ilorin, Nigeria; 13 African Centers for Disease Control and Prevention, Addis Ababa, Ethiopia; 14 Department of Psychiatry, Ahmadu Bello University Teaching Hospital, Zaria, Kaduna State Nigeria; 15 Department of Community Medicine, Ahmadu Bello University Teaching Hospital, Zaria, , Kaduna State Nigeria

**Keywords:** Monkeypox, Awareness, MPXD, Nigeria

## Abstract

The Monkeypox Disease (MPXD) gained attention due to its widened geographical distribution outside of Africa, Hence, a it was declared a global health emergency. The index case in Europe was from a Nigerian traveller. This study assessed public awareness and knowledge of the MPXD by conducting an online, cross-sectional survey of educated Nigerians. A total of 822 respondents were recruited using the snowball sampling method between the 16th to 29th of August 2022. More responses were retrieved from the Northeastern geopolitical region (30.1%, *n*=220) than other regions. Descriptive statistics revealed that 89% (*n*=731/822) of the study participants were aware of the MPXD but only 58.7% (*n*=429/731) of them had good knowledge of the disease with a mean knowledge score of 5.31±2.09. The main knowledge gaps were in the incubation period of the disease, the signs and symptoms, its mode of transmission, and preventive practices needed to curb the spread of the monkeypox virus (MPXV). Specifically, only 24.5% (*n*=179) of them knew that MPXV can be transmitted via sexual contact. Most of the study participants (79.2%, *n*=651) opined that we can prevent the occurrence of public health emergencies in the future. The multivariable logistic regression analysis revealed that of the socio-demographic variables, the male gender (OR: 1.69; 95% CI: 1.22,2.33); Ph.D. level education (1.44; 95% CI: 1.048,4.23); and being homosexual (OR:1.65; 95% CI: 1.07,3.78) were significantly associated with good knowledge of MPXD. Despite the varying prevalence across the country, the region of residence within Nigeria did not influence the knowledge of MPXD among the respondents. The knowledge gaps necessitate intensified public health risk communication with a focus on modes of transmission and the preventive measures needed to curb the spread of the MPXV.

**Supplementary Information:**

The online version contains supplementary material available at 10.1186/s12889-023-15398-0.

## Introduction

Monkey Pox Disease (MPXD), a re-emerging zoonosis with a close resemblance to smallpox; was declared a public health emergency on the 23^rd^ of July 2022. [1]. Before the 2022 outbreak, MPXD was considered a neglected tropical disease, and belonged to two main clades: Central- and West- African clades based on their region of occurrence in Africa [2, 3]. As a close relative of smallpox (caused by the variola virus), initial observations revealed that the monkeypox virus (MPXV) had a lower risk of human-to-human transmission, a lower secondary attack rate, and previous smallpox vaccinations appeared to be 85% protective in preventing the disease [4, 5]. Globally, the 2022 MPXD outbreak has been confirmed in 99 countries and has caused 50, 844 cases out of which 3,451 cases were reported in Africa as of 31^st^ August 2022 [6, 7].

Although the natural reservoir of MPXV remains unknown, several animal hosts (for example rodents and non-human primates) have been considered to be reservoirs of the MPXV, and increasing contact with these hosts could result in occasional spill-over and sporadic outbreaks in humans [8, 9, 10, 11].

Epidemiologically, several studies have hypothesized the source of infection and spread of the current outbreak. Studies have attributed the outbreak to unrecognized local chains of transmission from imported cases or mass gathering events that have multiplier effects [12, 13]. In a multinational study involving 16 countries, Thornhill et al. reported 528 infections that were diagnosed between April and June 2022 in which 98% of the infected persons were gay, 75% were White, and 41% had an underlying human immunodeficiency virus infection [14]. Clinically, 95% of infected patients presented with rashes, 73% with anogenital lesions, and 41% with mucosal lesions [15]. In other patients, the morphology of the rash does not progress from maculo-papules to blisters and pustules as in typical cases, instead, pustules have appeared before systemic symptoms in some cases [11, 16, 17].

In Nigeria, sporadic cases of MPXD were reported annually between 2017 to 2022 [5, 18, 19]. However, the cases had increased significantly in 2022 in which the country had 530 suspected cases with 220 confirmed cases from 29 out of the 37 states [19]. The number of confirmed cases likely represents a small proportion of the true number of infections, due to the majority of cases being asymptomatic, poor testing rate, cases not presented to health facilities, and incomplete reporting practices [10]. In addition, the impact of changes in land-use systems, deforestation, and lifestyle may influence the spillover and detection of pathogens such as MPXV [20–22]

With the declaration of MPXD as a global health emergency and the increasing cases in Nigeria, public awareness and knowledge of the disease could help identify knowledge gaps that could be crucial to its containment. Hence, we conducted an online survey to determine the knowledge of MPXD among educated Nigerians.

## Materials and methods

### Study design, study participants, sample size, and sampling

This study was conducted as an online cross-sectional survey of the general public irrespective of their age, background, or any socio-demographic attributes. The inclusion criteria were age (18 years and above), residence (Nigeria), and consent (willingness to participate in the study voluntarily). To calculate the sample size for this survey, we hypothesized that at a 96% confidence interval and a 4% margin of error, 50% of the respondents would have a satisfactory knowledge level of MPXD. Using these parameters, 660 responses were needed to give a good representation. However, 822 responses were received within this time frame. The study participants were recruited using a snowballing (convenience) sampling method by relying on our social media connections. Thus, respondents were recruited via social media platforms such as WhatsApp and Facebook Messenger. The survey was launched on the 16^th^ of August 2022 and the dataset used in this study was extracted on the 29^th^ of August 2022.

### Questionnaire design

To evaluate the awareness and knowledge of the MPXD among a cross-section of Nigerians, we designed a survey instrument and administered the structured pre-validated questionnaire using Microsoft forms (https://forms.office.com/r/SNmrCXPWmM). The questionnaire was pre-validated by two independent reviewers, and a pre-test study was conducted with 20 respondents from three of the six geopolitical zones of Nigeria. Furthermore, we assessed the reliability of the survey instrument using the Cronbach Alpha test and obtained a score of 0.73. The questionnaire consisted of 27 questions that were subdivided into four segments. While the first section obtained information on the demography of the respondents, section B assessed the awareness and knowledge of monkeypox disease. The two other sections (Sections C and D) obtained information relating to preventive practices and the respondents’ perspectives on the global health emergency respectively.

### Data analysis

The data were analyzed using Minitab v.17 (Pennsylvania, USA). The descriptive statistics were summarized as frequencies and proportions for qualitative variables whereas quantitative variables were presented as mean ± standard deviation. As previously described, we used a numeric scoring system to determine the knowledge of MPXD among respondents who were aware of the disease [23]. Briefly, a correct response attracted a score of 1, while an incorrect response attracted a score of 0. Note that for questions that have multiple correct answers, each correct response was awarded 0.125 or 0.25 based on the number of correct options in each question. Then, we computed the total of each respondent’s score and graded them as having ‘good” or ‘poor’ knowledge of MPXD based on a 10-item scale. The cut-off for this grading was 50% of the maximum obtainable score (*n* = 10 points). Hence, respondents who had a cumulative score of < 5 points were graded as having poor (inadequate) knowledge of MPXD whereas those who scored between 5–10 points were graded to have good (adequate) knowledge of MPXD. Finally, we used univariable and multivariable logistic regression analyses to test for all significant associations between the independent variables (e.g., age, sexual orientation, gender, level of education, and region of residence) and the outcome variable (knowledge of MPXD) at a *p* < 0.05 and a 95% CI, respectively.

## Results

### Respondent demographics

A total of 822 respondents were included in this online cross-sectional survey. More male respondents (57.4%, *n*=472) attempted the survey than female respondents (41.6%, *n*=342). All of the respondents were highly educated and had tertiary education with only 4.38% (*n*=36/822) having less than a university or college degree. Approximately 50.5% (*n*=415) of the study participants were heterosexual. Other sexual orientations among the study participants were bisexual (9.9%, *n*=81) and homosexual (5.6%, *n*=46) (Table 1). More responses were retrieved from the Northeastern geopolitical region (30.1%, n=220) whereas the least response was from the South Eastern region (2.9%, *n*=21).

**Table 1:** Demographics of study participants (*n* = 822)

Variables	Frequency (%)
**Age (Years)**	
18–29	220 (26.8)
30–39	390 (47.5)
40–49	165 (20)
50–59	38 (4.6)
> 60	9 (1.1)
**Gender**	
Female	342 (41.6)
Male	472 (57.4)
Non-binary	2 (0.24)
Prefer not to say	6 (0.73)
**Sexual orientation**	
Bisexual	81 (9.85)
Heterosexual	415 (50.5)
Homosexual	46 (5.6)
Prefer not to say	280 (34.06)
**Level of education**	
No formal education	0 (0)simpla
College/University degree	497 (60.46)
High School	36 (4.38)
Master/professional fellowships	243 (29.56)
PhD	44 (5.35)
**Geo-political region**	
North Central	170 (23.26)
North East	220 (30.10)
North West	84 (11.49)
South East	21 (2.87)
South South	91 (12.45)
South West	145 (19.84)

### Awareness and knowledge of monkeypox disease

**Fig. 1:**
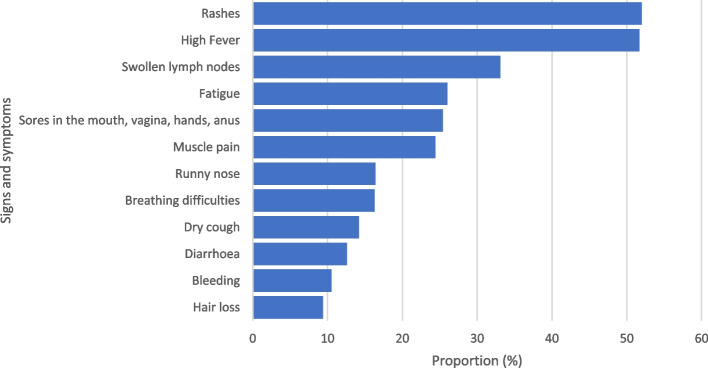
Awareness of signs and symptoms of MPX among study participants (*n* = 731)

Most of the study participants (89%, *n*=731/822) were aware of the 2022 MPXD outbreak. More information about the MPXD were obtained from internet sources (33.4%, *n*=244), social media platforms (28.6%, *n*=209), and TV (25.6%, *n*=187). The mean knowledge score of the respondents was 5.31±2.09 with a range of 0.125 to 9.375 from a maximum obtainable score of 10. Based on the cut-off of 5.0 (50% of the maximum obtainable score), more than half of the respondents (58.7%, *n*=429/731) had good knowledge of MPXD, whereas 41.3% of the respondents (*n*=302/731) were graded as having poor knowledge. One-third (33.9%, *n*=248) of the respondents knew the incubation period for MPXD was 5–21 days. Similarly, only 35.3% (*n*=258) of them knew that MPXD could be asymptomatic and patients could manifest no specific symptoms. Approximately two-thirds of the respondents (66.34%, *n*=485) knew that anyone irrespective of their age, gender, sexual orientation, or underlying condition could be affected by the MPXD. Of the varying symptoms, respondents were mostly aware of the prodromal symptoms such as high fever (51.7%, *n*=378), and fatigue (26%, *n*=190). Of the more specific MPXD symptoms, participants knew that rashes (52%, *n*=380), swollen lymph nodes (33.1%, *n*=242), and sores in the hands, mouth, vagina, and anus (25.44%, *n*=186) were associated with the MPXD (Fig. 1). On the modes of transmission of the MPXV, the study participants knew that the virus could be transmitted via contact with contaminated surfaces (32.83%, *n*=240), close contact with infected people (42.68%, *n*=312), and through sexual intercourse (24.48%, *n*=179). In addition, approximately one-quarter of the respondents (24.48%, *n*=179) knew that hunting and contact with wildlife could transmit the MPXV to humans. As preventive measures, respondents knew that vaccination against MPXD (46.37%, *n*=339), proper hygiene practices (46.1%, *n*=337), and practicing safe sex (21.75%, *n*=159) were important in reducing the spread of the MPXV and control the outbreak (Table 2).

**Table 2:** Knowledge of Monkey Pox Disease among respondents in Nigeria (*n*=822)

Variables	Frequency (%)
1. Have you heard of the 2022 monkeypox outbreak?	
No	91 (11)
Yes	731 (89)
1b. Source of information on MPXD?	
TV	187 (25.6)
Radio	94 (12.9)
Internet sources	244 (33.4)
Social media platforms	209 (28.6)
Newspaper	49 (6.7)
Friends/Family	54 (7.4)
WHO website	73 (10)
2. Are you aware the MPXD was declared a global health emergency?	
No	125 (17.10)
Yes	606 (82.90)
3. Can we have asymptomatic cases of the MPXD?	
No	178 (24.35)
Yes	258 (35.29)
I don't know	295 (40.36)
4. What is the incubation period for MPXD?	
1–14 days	89 (12.18)
1–3 months	16 (2.19)
5–21 days	248 (33.93)
I don’t know	378 (51.71)
5. Who can be infected with the MPXD?	
Anyone can be infected	485 (66.34)
Teenagers	12 (1.64)
People with chronic diseases	17 (2.32)
Only those who practice unsafe sex	35 (4.78)
Only old people	2 (0.27)
6. How does the virus spread?	
Air droplets	178 (24.35)
Mosquito/Insect bites	35 (4.78)
Contact with contaminated surfaces	240 (32.83)
Sexual intercourse	179 (24.48)
Close contact with people that have the virus	312 (42.68)
Hunting and contact with wildlife	179 (24.48)
Processing & consumption of inadequately cooked animal products	150 (20.52)
I don’t know	85 (11.63)
7. Which of these can be protective against MPX?	
Antibiotics	87 (11.90)
Proper hygiene such as handwash	337 (46.10)
Practicing safe sex	159 (21.75)
Vaccination against MPX	339 (46.37)
None of the above	2 (0.27)
I don’t know	55 (7.52)
8. How important is hand hygiene in reducing the spread of viruses (monkeypox virus)?	
Not important	422 (57.72)
Averagely important	175 (23.93)
Extremely important	134 (18.33)

### Perception of the monkeypox disease

Despite the high educational qualifications, less than half of the respondents (46.47%, *n*=382) knew that the MPXV can be transmitted via sexual intercourse. Only 72% (*n*=592) of the respondents were sexually active and 85.47% of them (*n*=506/592) believed that they practice safe sex (Table S1). Eighty percent of the respondents (*n*=661/822) followed the recommendations of the nation’s health agencies but only 36% of them (*n*=238/661) followed all of the recommendations. Only 4.99% of the respondents have been vaccinated against monkeypox. One-third of the study participants (33.82%, *n*=278) opined that their health authorities either at the federal or sub-national levels were doing enough to safeguard their health. Most of the study participants (79.2%, *n*=651) opined that we can prevent the occurrence of public health emergencies in the future. Specifically, they believed that raising public awareness of proper hygiene (37.83%, *n*=311), establishing early alert systems for infectious diseases (36.74%, *n*=302), as well as improving surveillance in the human and animal health sectors (35.52%, *n*=292) are critical to preventing or delaying the emergence of the next global health emergency (Table 3).

**Table 3:** Perception of community and global response to the 2022 monkeypox outbreak ($n=822$)

Variables	Frequency (%)
1. Do you think that your health authorities are doing enough to safeguard your health?	
Maybe	239 (29.08)
No	305 (37.10)
Yes	278278 (33.82)
2. Do you think we can prevent such a global pandemic in the future?	
Maybe	141 (17.15)
No	30 (3.65)
Yes	651 (79.20)
2b. if yes, how?	
Reduce international travels	88 (10.70)
Establish early alerts and global warning systems for infectious diseases	302 (36.74)
Improve surveillance in the human and animal health sectors	292 (35.52)
Intensify research on preventive measures such as vaccines/ diagnosis	304 (37)
Raise public awareness of proper hygiene/healthy habits	311 (37.83)
Prioritize safe sex practices	177 (21.53)
3. How satisfied are you with the social media coverage of the Monkeypox Outbreak?	
Least satisfied	88 (10.7)
Satisfied	156156 (18.97)
Average	265 (32.2)
Very satisfied	147 (17.9)
Extremely satisfied	166 (20.2)

### Impact of the socio-demographic factors on the knowledge of monkey pox disease among study participants

Three of the five socio-demographical factors were significantly associated with good knowledge of the MPXD. Our analysis revealed that male respondents were more likely to have good knowledge of MPXD than their female counterparts (OR: 1.69; 95% CI: 1.22,2.33; *p*=0.001). Similarly, adequate knowledge of MPXD was positively associated with the increasing level of education. Hence, respondents with a Ph.D. were more likely to have more knowledge of MPXD than other educational categories (OR: 1.44; 95% CI: 1.04,4.23; *p*=0.05). Finally, the sexual orientation of the respondents was significantly associated with the knowledge of MPXD as respondents who were homosexuals were more likely to have better knowledge of the disease than other sexual orientations (OR:1.65; 95% CI: 1.07,3.78; *p*=0.049). However, the age, as well as the residence (geo-political region), did not influence the knowledge of MPXD among the residents (Table 4).

**Table 4:** Analysis of sociodemographic variables as factors influencing the public knowledge of respondents towards the 2022 monkey pox outbreak

Variables				Univariable analysis		Multivariable analysis	
		Satisfactory (%)	Unsatisfactory (%)	OR (95% CI)	*P*-value	AOR (95% CI)	*P*-value
Age (years)	18 – 29	91	82	1.00	-	-	-
	30 – 39	212	152	1.26 (0.87, 1.82)	0.001	1.07 (0.71,1.61)	0.673
	40 – 49	104	51	1.82 (1.16, 2.85)		1.28 (0.76,2.16)	
	50 – 59	22	17	1.16(0.57, 2.34)		0.87 (0.40,1.91)	
Gender	Female	159	144	1.00	-	-	-
	Male	267	154	1.61 (1.19, 2.18)	0.002	1.69 (1.22,2.33)	0.001
Sexual orientation	Bisexual	39	30	1.00	-	-	-
	Heterosexual	243	137	1.37 (0.82, 2.32)	0.003	1.37 (0.79,2.34)	0.049
	Homosexual	27	14	1.48 (0.66, 3.30)		1.65 (1.07,3.78)	
	Prefer not to say	120	121	0.76 (0.44, 1.30)		0.85 (0.48,1.50)	
Education	High School	16	12	1.00	-	-	-
	College (Bachelor)	240	199	0.91 (0.42, 1.96)	0.047	1.00 (0.45,2.25)	0.05
	Masters	146	80	1.37 (0.62, 3.05)		1.22 (0.52,2.89)	
	Ph.D	27	12	1.68 (0.61, 4.64)		1.44 (1.04,4.23)	
Geopolitical region	North East	114	106	1.00	-	-	-
	North Central	100	70	1.34 (0.89, 2.00)	0.015	1.19 (0.78,1.84)	0.155
	North West	46	38	1.11 (0.67, 1.86)		1.08 (0.64,1.84)	
	South East	16	5	3.00 (1.06, 8.48)		2.86 (0.99,8.29)	
	South South	65	26	2.27 (1.34, 3.85)		1.86 (1.05,3.31)	
	South West	90	55	1.52 (0.98, 2.34)		1.26 (0.79,1.99)	

*OR* Odd’s ratio, *AOR* Adjusted Odd’s ratio

## Discussion

The 2022 MPX outbreak among other zoonoses has once again highlighted the importance and inter-relatedness between human, animal, and environmental health and highlights the need for investment and strengthening of healthcare systems to limit spillover events. Furthermore, these zoonotic diseases have no borders, especially with globalization and increased travel [22, 24]. Therefore, controlling them requires a One Health approach [25].

In Nigeria, there are knowledge gaps on the epidemiology, animal reservoir, role of environmental factors and domestic animals in MPXV transmission, genetic diversity of the MPXV, and public awareness of the disease [2, 22, 24, 25]. From our study, the awareness of MPX was high among the study population. This could be due to the declaration of the disease as a public health emergency of international concern (PHEIC), the high education level of the respondents, and several online public health advisories on MPX. Despite the high awareness rate, only 58.7% of them had a good knowledge of the incubation period, symptoms, route of transmission, and preventive practices that could be used to limit the spread of the MPXD. The observed knowledge gaps were mostly in the symptoms and the non-awareness that most cases of MPXD could be asymptomatic and patients could manifest no specific symptoms. In addition, asymptomatic cases of MPX have been reported in attendees of a male sexual health clinic in Belgium after retrospective samples were screened for MPXV [26]. In Nigeria, it was reported that the rate of asymptomatic MPX infection could be as high as 28% [5].

Another key knowledge gap is in the awareness that the MPXV can be transmitted via sexual contact as our findings showed a low awareness of sexual contact as a mode of transmission of the MPXV. Globally, there have been discussions on whether MPX infections should be regarded as a sexually transmitted disease (STD). Although the MPXV can be transmitted via close contact, including sexual contact, it is not a sexually transmitted disease (STD) [27, 28]. This low awareness of the sexual transmission of the MPXV could cause poor risk perception of the disease and increased human-to-human transmission. To reduce the global burden of the MPXD, Spicknall et al. reported that a 40% reduction in one-time sexual partnerships might delay the spread of monkeypox and reduce the percentage of persons infected by 20–31% [29]. In addition, Raccagni et al. reported that MPXV was detected in 61% of the individuals with confirmed cases of MPX through blood testing. Hence, reducing sexual partnerships and contacts would significantly reduce the MPXD burden [30].

Although most of our study participants knew that anyone can be infected with the MPXV, a few of them thought that only persons that practice unsafe sex or those with underlying conditions could be affected. This could be attributed to the fact that most human-to-human cases in the international clusters had an underlying condition, especially the human immunodeficiency virus (HIV/AIDS) [27, 31]. Furthermore, Raccagni et al. reported that 84% of individuals with MPX were either HIV Pre-Exposure Prophylaxis Users (PrEP) or were people living with HIV (PLWH) [30]. In another study, Raccagni et al. reported a case of MPX infection together with a pan-resistant *Campylobacter* sp. infection and an *Entamoeba histolytica* and *Chlamydia trachomatis* proctitis re-infection [32]. In Nigeria, it was previously reported that most of the MPX-positive persons had HIV with features of AIDS [5].

The knowledge of correct preventive measures against MPXV among our respondents was below average. This is evident in the fact that less than half of the respondents recruited into this study knew that vaccination against MPXD, proper hygiene practices, and avoiding contact with infected persons and wildlife could limit the spread of most pathogens including MPXV. This is important as it is widely believed that most identified cases of MPXD in Nigeria were from presumed animal exposure. However, it remains unclear whether most of the 2022 MPX cases in Nigeria were from contact with wildlife or through human-to-human transmission. Earlier, few cases have been reported due to human-to-human transmission from a Nigerian prison [5].

The ongoing COVID-19 pandemic had several impacts on the control of other infectious diseases such as MPX. This included the poor detection and reporting of MPX cases in Nigeria during the COVID-19 pandemic [18]. The poor perception of the government’s handling of the COVID-19 pandemic could also affect the perception of the 2022 MPX outbreak. This could explain why only one-third of our study participants opined that Nigeria’s health authorities were doing enough to safeguard their health and only 36% followed all of the recommendations and public health advisories of the NCDC. Finally, it was encouraging that the perception of respondents on the possibility to prevent, limit, or delay the next global health emergency was good. For instance, they believed that establishing early alert systems for infectious diseases, improving surveillance in the human and animal health sectors, reducing international travel, and minimizing human footprint on the environment amongst others could help delay the emergence of pathogen X (the next global health emergency).

Our logistic regression analysis showed that some of the socio-demographic variables were positively associated with good knowledge of MPXD. Therefore, respondents with a Ph.D. (level of education), male respondents (gender), and those that were homosexual (sexual orientation) were more likely to have good knowledge of MPX than others. While Ph.D. education could be directly associated with more research and information gathering resulting in better knowledge, it remains unclear to us why male respondents had better knowledge of MPX. With a novel route of transmission (sexual contact), the higher knowledge of MPX among bisexuals could be due to their increased risk perception and the need to protect themselves. Even for regions that are MPX hotspots in Nigeria, there were no significant differences in the knowledge level of their respondents. This could be due to uniform public health advisories across Nigeria and the impact of non-restricted information on social media platforms that are available across the country.

Several factors could be responsible for the re-emergence of zoonotic diseases such as MPX in Africa. These include the poor animal health surveillance system, waning immunity against smallpox which offered cross-protection for MPXV, the masking effect of the COVID-19 pandemic, increased contact with wildlife, especially with population explosion, the capture, processing, sale, and consumption of game animals, as well as poor diagnostic infrastructure in resource-limited settings as the reported number of cases, are only the tip of the iceberg [5, 21, 24]. Therefore, investments in integrated surveillance at the human-animal-ecosystem interface using a One Health approach could be the key to curbing the spread of the MPXV [22, 33]. This is even more important now as there is evidence of human-to-animal (dog) reverse transmission of the MPXV [34].

The main limitation of the study is the non-randomness of the study participants, which makes the *p*-value not very reliable. Hence, the findings of this survey might not be used for making inferences about the general Nigerian population. On the contrary, online surveys avoid some contextual social desirability biases such as the sexual orientation of the respondents.

## Conclusion

The 2022 MPX outbreak has caused a global health emergency with confirmed cases from 99 countries. The index case in Europe was from a Nigerian. The analysis of our findings showed a high awareness of MPX but an average knowledge of the disease. The main gaps were in the mode of transmission, signs and symptoms, and the preventive measures needed to curb the spread of the MPXV. In addition, the level of education (Ph.D.), gender (male), and sexual orientation (Bisexual) of the respondents were associated with good (adequate) knowledge of MPXD. To curtail the spread of the MPXV, Nigeria must improve its integrated disease surveillance system using a one-health approach.

## Supplementary Information

**Additional file 1: Table S1. **Perception of monkeypox among study participants (*n*=822).

## Data Availability

The survey instrument and dataset are available as supplementary data.
