# Supplementary material for: A cross-sectional survey of public knowledge of the monkeypox disease in Nigeria
Source: BMC Public Health. 2023 Mar 29;23:591. doi: 10.1186/s12889-023-15398-0 (PMC10054201; doi:10.1186/s12889-023-15398-0)
Supplement: Supplementary file 1 — Additional file 1: Table S1. Perception of monkeypox among study participants (n=822). [file 12889_2023_15398_MOESM1_ESM.docx]

**Supplementary file**

**Table S1.** Perception of monkeypox among study participants (n=822).

| Variables | Frequency (%) |
| --- | --- |
| 1. Do you know that the MPX virus can be transmitted via sexual intercourse? |  |
| I don't know | 210 (25.55) |
| No | 230 (27.98) |
| Yes | 382 (46.47) |
| 1. Are you sexually active? |  |
| No | 230 (28) |
| Yes | 592 (72) |
| 1. Do you practice safe sex? |  |
| Maybe | 39 (6.59) |
| No | 47 (7.94) |
| Yes | 506 (85.47) |
| 3b. If yes, how? |  |
| Abstinence | 102 (20.15) |
| Sticking to my partner only | 411 (81.22) |
| Condoms | 484 (95.65) |
|  |  |
| 1. Do you follow the recommendation of your national health agencies? |  |
| Maybe | 103 (12.53) |
| No | 58 (7.06) |
| Yes | 661 (80.41) |
| 1. To what extent do you follow these recommendations? |  |
| I follow all the recommendations | 238 (36.01) |
| I follow most of them | 296 (44.78) |
| I follow some but not all | 118 (17.85) |
| Not at all | 9 (1.36) |
| 1. Have you been vaccinated against monkeypox? |  |
| No | 781 (95.01) |
| Yes | 41 (4.99) |
